# The Role of Discrimination in Social Safety and its Interplay with Adolescents’ Mental Health and Substance Use: A Network Perspective

**DOI:** 10.1007/s10964-026-02344-7

**Published:** 2026-03-30

**Authors:** Hanan Bozhar, Susanne R. de Rooij, Anja Lok, Tanja G. M. Vrijkotte, Reinout W. Wiers, Helle Larsen

**Affiliations:** 1 Dept. of Public and Occupational Health, Amsterdam University Medical Center, University of Amsterdam, Van der Boechorststraat 7, 1081 BT Amsterdam, Netherlands; 2 Centre for Urban Mental Health, University of Amsterdam, Oude Turfmarkt 147, 1012 GC Amsterdam, Netherlands; 3 Amsterdam Public Health Research Institute, Amsterdam University Medical Centers, Amsterdam, Netherlands; 4 Dept. of Epidemiology and Data Science, Amsterdam University Medical Center, University of Amsterdam, Meibergdreef 9, 1105 AZ Amsterdam, Netherlands; 5 Amsterdam Reproduction and Development Research Institute, Amsterdam University Medical Centers, Amsterdam, Netherlands; 6 Dept. of Psychiatry, Amsterdam University Medical Centers (Amc), University of Amsterdam, Meibergdreef 5, 1105 AZ Amsterdam, Netherlands; 7 Addiction, Development and Psychopathology (ADAPT)-lab, Dept. of Psychology, University of Amsterdam, Nieuwe Achtergracht 129-B, 1018 WS Amsterdam, Netherlands

**Keywords:** Discrimination, Social safety, Urban neighborhood, Adolescents, Mental health, Substance use

## Abstract

Discrimination, as experienced by adolescents, represents a major societal issue with implications for mental health and risky health behaviors. Yet the interplay between these factors in the context of social safety in urban living environments remains insufficiently understood and was explored in the current study. Participants were 1,675 Dutch adolescents (*M*age = 17.44 years, *SD* = 0.36; 56.6% girls) who participated in the ABCD-study. This subjective data was combined with objective neighborhood indicators from the GECCO‑consortium. Cross-sectional network analyses revealed discrimination, urban hassles, and sense of neighborhood security as central nodes connecting objective neighborhood social safety with anxiety/depression symptoms and substance use. Discrimination was negatively associated with sense of security and alcohol use; but positively with urban hassles experiences, which was directly related to higher levels of depressive symptoms and cannabis use. Everyday discrimination was indirectly related to mental health through reduced well-being and weaker social support, and directly linked to urban hassles and cannabis use. The significantly higher global strength in girls may suggest heightened sensitivity to discrimination and related stressors. Highlighting the importance of discrimination in adolescents’ perceptions of neighborhood social safety and its connections with psychological distress, these findings call for longitudinal research to clarify pathways within residential environments.

**Supplementary Information:**

The online version contains supplementary material available at 10.1007/s10964-026-02344-7.

## Introduction

Adolescents’ mental health is shaped not only by individual vulnerabilities but also by the social and environmental conditions in which they grow up (Branje et al., 2021; Bronfenbrenner & Morris, 2007). Experiences of discrimination and perceptions of neighborhood social safety are increasingly recognized as important social stressors (e.g., Benner et al., 2018; Bozhar et al., 2025; Cave et al., 2020), yet their combined role in adolescents’ psychological distress and substance use remains insufficiently understood. Existing research has typically examined discrimination, environmental stressors, and neighborhood disadvantage separately, limiting insight into how these factors jointly operate in adolescents’ daily lives. This study addresses this gap by examining how discrimination interrelates with objective and subjective indicators of neighborhood social safety, mental health, and substance use in late adolescence.

### Social Context and Safety in Adolescence

Mental health problems in late adolescence (age 17–18) represent a significant public health issue, not only due to their immediate impact on well-being but also because they are associated with long-term consequences that extend into emergent adulthood (Arnett, 2024). The social environment is fundamental to mental health, serving as both a protective and risk mechanism (Midouhas et al., 2021; Visser et al., 2021). Supportive social environments, characterized by positive relationships, access to educational, financial, and emotional resources, and a sense of stability in daily life and future prospects, can buffer against stress and promote resilience (Breedvelt et al., 2022; Kingsbury et al., 2020). Conversely, adverse social conditions such as poverty, exposure to violence, or lack of social support can exacerbate effects of stress, undermine coping mechanisms, and contribute to the onset or worsening of mental health problems (Kingsbury et al., 2020; Martin et al., 2019). This is especially the case in older or late adolescents, as this developmental stage involves increased autonomy, identity formation, and exposure to adult-like responsibilities, heightening sensitivity to the social environment (Arnett, 2024; Branje et al., 2021). In a large population-based birth cohort, previous analyses demonstrated that objectively measured neighborhood social safety, perceptions of the living environment, and sense of security appeared especially important for late adolescents’ mental health and substance use (Bozhar et al., 2025). The sense of safety within communities may not only be determined by actual crime rates or external threats but also by subjective perceptions of security, such as urban hassles. Urban hassles—such as safety concerns, coercion, and environmental nuisance—are recurring stressors in urban settings that may disrupt adolescents’ sense of security and contribute to stress and psychological complaints (Miller & Bennett, 2016). These experiences are particularly relevant in densely populated areas, where exposure to drug-related nuisance or street disturbances may amplify feelings of vulnerability and social stress.

### Discrimination: A Social Stressor?

Another potential factor that may affect feelings of safety is perceived discrimination (hereafter discrimination), which refers to the subjective experience, belief, or expectation of being treated negatively, unfairly, or with prejudice due to personal characteristics such as ethnicity, gender, age, socioeconomic position, or other social identities (Boer et al., 2022; Dai et al., 2024; Mello, 2024). In the Netherlands, adolescents report twice as many experiences of discrimination compared to older adults (65 + ) (Andriessen et al., 2020). A report by the Amsterdam Children’s Ombudsman (2024) highlighted that local adolescents regularly experience racism and unfair treatment in daily settings by both peers and adults, while a UNICEF (2023) national survey found that nearly half of adolescents have witnessed discrimination among peers and perceive it as a major societal issue. Recent national reports show that Dutch adolescents most often encounter discrimination based on ethnicity (63%), skin color (52%), and religion (48%), followed by gender (17%) and sexual orientation (12%) (Day & Badou, 2019). Such experiences occur across social contexts such as schools, sports clubs, and public spaces, and are especially common among youth with Moroccan, Turkish, and Surinamese backgrounds. Already in 2009, a meta-analysis showed that increased levels of discrimination were associated with more negative mental and physical health outcomes (Pascoe & Smart Richman, 2009). In adolescents, greater perceptions of discrimination have also been found to be related to psychological complaints such as depressive and internalizing symptoms [meta-analysis:(Benner et al., 2018)] and reduced well-being (Priest et al., 2013). Recent findings further indicate that discrimination related to race or ethnicity predicts both current and future levels of depressive symptoms, as well as the subsequent onset of alcohol use (Bo & Jaccard, 2024). Feeling discriminated against may amplify feelings of vulnerability and social exclusion, which has been associated with mental health problems up to 2–6 years after exposure to discrimination (Lei et al., 2021). Adolescents experiencing discrimination may also be more likely to engage in substance use behaviors (Dai et al., 2024; Gerrard et al., 2012). Over time, these experiences may contribute to the development of mental health conditions and subsequent problematic substance use (Lavner et al., 2022; Lei et al., 2021). This is particularly concerning for late adolescents, who rely on a stable social environment as they navigate increasing responsibilities and the challenges of identity formation (Branje et al., 2021; Bronfenbrenner & Morris, 2007). Discrimination may act as a chronic social stressor, triggering emotional distress and physiological stress responses that increase vulnerability to maladaptive coping strategies, such as substance use (Cave et al., 2020; Gerrard et al., 2012). According to the self-medication hypothesis, adolescents may engage in substance use to alleviate the negative affect associated with repeated experiences of exclusion, hostility, or social devaluation (Lavner et al., 2022). Hence, the link between discrimination and substance use may occur both directly through stress-coping pathways and indirectly through co-occurring mental health problems. Also, the cumulative burden of discrimination may undermine the protective benefits of a safe and supportive community. Adolescents’ perceptions of safety may be disrupted, as discrimination may introduce a recurring sense of exclusion, distrust, or hostility within their social context (Cave et al., 2020; Priest et al., 2013), affecting various domains of daily life (Zhao et al., 2024).

While several studies have demonstrated associations between discrimination, neighborhood disadvantage, and adolescent well-being (e.g., Benner et al., 2018; Trucco et al., 2023; Visser et al., 2021), most of this work has been conducted outside the European context and focused on school or community settings rather than adolescents’ residential environments. Moreover, these studies often considered discrimination and environmental stressors as separate risk factors. A systematic and more complex approach examining the different layers of environmental influences related to mental health and substance use in late adolescents is missing. Network analysis is a suitable approach as it provides a systematic perspective on how variables across domains—mental health, social safety, substance use, and neighborhood context—are interrelated in adolescents, but has not been applied in the context of discrimination, environmental stressors and adolescent well-being. This approach moves beyond isolated associations and reveals key bridging variables that may serve as targets for further research and intervention. It also highlights structural differences in how these domains are connected across subgroups, offering insights not captured by traditional regression-based methods. Unlike these methods, which focus on latent constructs and directional pathways, network analysis allows for a detailed exploration of how individual variables—such as discrimination, urban hassles, and substance use—interact as part of a system. This approach offers a holistic view of adolescent well-being by identifying central and bridging variables that connect psychosocial domains, rather than treating them in isolation. Two other frameworks that are useful in the context of examining discrimination, environmental stressors and adolescent well-being are complexity modelling and ecological mental health models. Complexity-based models of mental health view psychological outcomes as emergent properties of dynamic systems involving biological, psychological, and environmental factors (Borsboom, 2017; Crielaard et al., 2021), while ecological models of adolescent development highlight the importance of a layered perspective on environmental influences—such as neighborhood safety and discrimination—linking mental health and behavioral outcomes (Bronfenbrenner & Morris, 2007).

## Current Study

There is a lack of studies applying a holistic, systems-based approach to studying the interplay between discrimination, environmental stressors, and mental health and substance use among adolescents, especially in the context of their residential environments in a European context. The current study aimed to address this research gap by investigating the interplay between depressive and anxiety symptoms, use of alcohol, tobacco and cannabis and discrimination, as well as objective and subjective neighborhood social safety in 17 to 18-year-old Dutch adolescents applying network analysis. To get a comprehensive understanding of social safety both objective and subjective measures of neighborhood safety is used as this can capture both the actual conditions and the personal experiences, which may vary even within similar objective conditions. The study is grounded in both complexity and ecological mental health models. Given the exploratory nature of this study, no predefined hypotheses were formulated. The guiding research question was: What is the role of discrimination in adolescents’ sense of neighborhood social safety, and how does this relate to mental health and substance use?

## Methods

### Population Sample and Procedure

We used data from the sixth wave of the Amsterdam Born Children and their Development study (ABCD-study). This longitudinal study started in 2003-2004 when all pregnant women living in Amsterdam were invited to participate in the cohort, and approximately 8000 children subsequently enrolled (van Eijsden et al., 2011). Wave 6 involved a questionnaire assessment, conducted in the period June-September 2021, when the participants were 17–18 years old. This wave was specifically designed to capture the unique aspects of late adolescence, focusing on mental health outcomes, well-being, living environment, and behaviors. Data was collected through digital (mobile app or weblink) or paper questionnaires. Completing the questionnaire took approximately 50 min, with the possibility to fill it out in multiple takes (6 thematic parts). Participants received a web shop voucher worth €10 upon completing the questionnaire. Some participants did not complete the full questionnaire. Consequently, 99 participants with missing data on discrimination, depressive symptoms, anxiety symptoms, or substance use and three participants with missing values on objective poverty scores were excluded. Little’s MCAR test supported the assumption of random missingness; therefore, listwise deletion was applied. This resulted in a final analytical sample of *N* = 1675 (56.6% women; Mage = 17.44 (*SD* = 0.36)). A sub-sample of participants who reported experiencing discrimination consisted of *N* = 205. Prior to participation, adolescents received detailed information about the study procedures and provided written informed consent themselves in accordance with ethical guidelines. As the adolescents were older than 16 years, parental consent was not required at this stage. The study protocol was approved by the Medical Ethics Review Committee of the Academic Medical Center Amsterdam (W20_396#20.444) as part of the ABCD cohort.

#### The Dutch Context

The Netherlands is a high income country with a relatively liberal social and policy climate, for example regarding diversity and gender norms (Hughes et al., 2023). Around 28% of the Dutch population has a migration background, reflecting substantial ethnic and cultural diversity (Statistics Netherlands, 2024). Norms related to family life, gender roles, and health behaviors differ across ethnic and migration-background groups in the Netherlands. Dutch adolescents generally grow up in a context that emphasizes individual autonomy and open discussion of sensitive topics. Attitudes toward alcohol, tobacco, and cannabis are comparatively tolerant, although national prevention policies increasingly promote healthy lifestyles (Defoe et al., 2023; Nuijen et al., 2023). These contextual factors are important for understanding behavior, self-perception, and group differences observed in this study.

### Mental Health

#### Depressive Symptoms

Depressive symptoms were assessed using The Patient Health Questionnaire (PHQ-9) (Kroenke & Spitzer, 2002), a self-report instrument consisting of nine questions related to different aspects of depressive symptoms. Participants were asked to rate the occurrence of each symptom over the past two weeks, where the response options ranged from 0 (“not at all”) to 3 (“almost every day”). The total score of the PHQ-9 ranges from 0 to 27. The internal reliability in the current study was good, with a Cronbach’s Alpha (α = 0.88).

#### Anxiety Symptoms

Anxiety symptoms were assessed using the 7-item General Anxiety Disorder-scale (GAD-7) (Spitzer et al., 2006). Similarly, the response options to indicate the severity of experienced anxiety complaints ranged from 0 (“not at all”) to 3 (“almost every day”). The total score of the GAD-7 ranges from 0 to 21. The internal reliability in the current study was good, with a Cronbach’s Alpha (α = 0.88).

#### Well-being

Well-being was assessed using Cantrils’ Ladder (Levin & Currie, 2014), to indicate overall life satisfaction, where participants rated their lives on a scale from 1 to 10, with 1 representing the worst imaginable life and 10 the best.

#### Stress

Stress was assessed by asking participants how often they experienced stress or tension in the past month, with response options ranging from 1 (“not at all”) to 4 (“three times or more per week”) and to what extent they experienced stress from social relationships (i.e. friends) in the past 12 months, with response options ranging from 1 (“none”) to 4 (“a lot”) (Keller et al., 2012).

### Substance Use (Last 3 Months)

#### Alcohol Use

Alcohol use was assessed by asking how often the participant had consumed alcohol, with response options ranging from 1 (“not”) to 9 (“daily”).

#### Tobacco Use

Tobacco use was assessed by asking how often the participant had smoked cigarettes/cut tobacco, ranging from 1 (“once or less in last 3 months”) to 6 (“daily”).

#### Cannabis Use

Cannabis use was assessed by asking how often the participant had ever used hashish/weed, ranging from 1 (“not”) to 6 (“more than 19 times”).

Participants who indicated to have never consumed alcohol, tobacco, or cannabis were assigned a zero accordingly.

### Social Safety Perceptions

#### Discrimination

Discrimination was assessed by asking participants whether they had ever felt discriminated against, with a yes/no response. If yes was responded, it was followed up by a question about the perceived motive for the discrimination. Participants could choose from multiple predefined categories, including sexual orientation, ethnicity, clothing, physical appearance, religion, and prefer not to say or another reason [open response]. To assess the recurrence of the experienced discrimination, the 9-item Everyday Discrimination Scale (EDS) (Williams et al., 1997) was used, which evaluates participants’ perceptions of routine experiences with unfair treatment in day-to-day life. Response options ranged from 1 (“Never”) to 5 (“Almost Always”). Example items include: “You are treated with less courtesy than other people” and “You are treated as if you are not smart”. Higher scores indicate a greater frequency of discrimination experiences in daily life. The total EDS score ranges from 9 to 45. The internal reliability in the current study was good, with a Cronbach’s Alpha (α = 0.83).

#### Sense of Security

Sense of security was assessed by asking participants if they feel secure in the neighborhood they currently live in, with response options ranging from 1 (“totally disagree”) to 5 (“totally agree”).

#### Social Contact

Social contact was assessed by asking participants with how many people from their neighborhood they have contact, with response options ranging between 1 (“I do not talk to anyone from my neighborhood”), 2 (“I have contact with 1 to 3 people from my neighborhood”), 3 (“I have contact with 4 to 7 people from my neighborhood”), and 4 (“I have contact with more than 8 people from my neighborhood”).

#### Social Support

Social support was assessed using the 12-item Multidimensional Scale of Perceived Social Support (MSPSS) (Zimet et al., 1988), with response options ranging from 1 (“Strongly Disagree”) to 7 (“Strongly Agree”). The items assessed participants’ perceptions of their social support network, including support from family, friends, and significant others (e.g., “I can talk with my friends about my problems” and “My family helps me make decisions”). The total score of the MSPSS ranges from 21 to 84. The internal reliability in the current study was good, with a Cronbach’s Alpha (α = 0.85).

#### Urban Hassles

Urban hassles were assessed using the Urban Hassles Index (Miller & Bennett, 2016) which consists of 16 items specifically designed to evaluate stressors that are distinctive to urban adolescents. Participants were asked to indicate how often they experienced these stressors over the past three months, ranging from 0 (“never”) to 3 (“very often”). Due to a technical mistake the last question of the UHI-16 was not included (item “waited for a bus near a dirty, smelly bus stop”, belonging to subscale “nuisance”), which resulted in 15 items. The items entailed questions indicating safety concerns, coercion, environmental nuisance, and drug-related nuisance (e.g., “being asked to sell/hide drugs” and “bothered by people hanging on streets”). The total score of the UHI ranges from 0 to 45. Higher scores indicate more experiences of daily hassles in the living environment. The internal reliability in the current study was good, with a Cronbach’s Alpha (α = 0.73).

### Objective Neighborhood Social Safety

Objectively measured neighborhood exposure data from various sources were provided by the Geoscience and Health Cohort Consortium (GECCO) (Lakerveld et al., 2020; Timmermans et al., 2018), and merged with ABCD data based on participants’ home addresses in the Netherlands. Specifically, data on neighborhood safety, social cohesion, and poverty were used. Positive values for *safety* and *social cohesion* correspond to higher levels of these dimensions compared to the national average in 2020 (z-scores).

#### Objective Social Cohesion

In line with the GECCO framework, social cohesion was operationalized as a composite indicator reflecting household diversity within a 300-meter radius (Herfindahl index), population density, mobility rate, and household development. These structural neighborhood characteristics are considered proxies for opportunities for social interaction and community stability. Perceived social cohesion was derived from the Dutch Safety Monitor, which includes items on trust, mutual help, and connectedness among residents. Although neighborhood interactions can vary in quality, the Safety Monitor items specifically capture positive aspects of connectedness, which are treated as indicators of cohesion. All indicators were combined using principal component analysis to generate a single objective social cohesion score.

#### Objective Safety

Safety was assessed through registered crime data, including categories such as burglary, theft, violent crimes, vandalism, and public order disturbances. This data was sourced from the Police’s Basic Information System (BVI), which records crimes linked to a report, victim, or suspect. Crime counts were spatially aggregated to the PC6 level, with additional weighting applied. Perceived safety was measured through the Safety Monitor, which includes survey responses on sense of security in the neighborhood. These data were combined and analyzed using principal component analysis to create an overall safety score for each neighborhood.

#### Objective Poverty

Poverty was assessed as the percentage of households in 2021 with an income at or below 101% of the national social minimum. This measure was derived from the Poverty Monitor, which links municipal financial support data with CBS data (Statistics Netherlands). The poverty data spans various income levels across different years, accounting for both current income status and long-term income status over the past four years.

### Socio-Demographics

The assessed demographics included participants’ biological *sex* (boy/girl) and *ethnicity* based on the maternal grandmother’s country of birth (Dutch; Surinamese, Antillean, Turkish, Moroccan, Ghanaian, Other Western, or Other Non-Western).

### Design and Data Analysis

First, the sample demographics were described (e.g., means, standard deviations, frequencies, percentages, and t-tests). Subsequently, a cross-sectional undirected network analysis was conducted (Epskamp et al., 2018), specifically using Mixed-Graphical-Modelling (MGM) as the data consisted of a combination of scale and categorical variables (Haslbeck & Waldorp, 2020). This network approach enables examining how individual variables are interrelated rather than viewing each variable in isolation. Each variable of interest represents a specific node within the network, and the edges in-between represent pairwise conditional associations similar to partial correlation coefficients.

Centrality measures identify the most influential or connected points in the network, highlighting which variables play a key role in the system as a whole. Bridge nodes are points in the network that serve as connections between different groups or clusters of variables. Identifying the primary bridge node within this network provides insight into how psychological health measures may be interconnected with the surrounding social environment, potentially highlighting key pathways. All analyses were conducted using RStudio (version 4.3.2).

#### Estimating the Networks

The network-models were estimated with the *bootnet*-package and visualized with the *qgraph*-package (Epskamp et al., 2018). Two networks with 17 nodes were estimated: a full-sample network including the dichotomous discrimination node (no/yes) and a sub-sample network limited to participants reporting discrimination, where the everyday discrimination score replaced the dichotomous node (adjusted for sex). Since MGM displays all edges with categorical nodes as positive, the sign of relevant edges with the dichotomous discrimination node were defined using the *ShowInteraction*-function (Haslbeck & Waldorp, 2020).

#### Node Strength

To quantify the influence of each node, centrality metrics were analyzed, including a measure of node *strength centrality*. This metric computes the weighted number and strength of all edges of specific nodes. Additionally, *bridge centrality* was examined (Jones et al., 2021) and clusters of closely connected nodes were explored. This identifies the nodes that are most strong in bridging important clusters within the network.

#### Assessing Model Stability

To assess the accuracy and stability of the model, non-parametric bootstrapping and case-dropping bootstraps were performed accordingly.

#### Comparing Networks: Boys vs. Girls

As “sex” appeared to be a central node in the networks, the full-sample network (higher stability) was examined for potential differences between boys and girls. A permutation test was performed using the *NetworkComparisonTest*-package (van Borkulo et al., 2023) to assess potential differences in global strength, network structure, and edge strength between the groups. The global strength invariance hypothesis implies that the total sum of edge weights, describing the total connectivity within a network, is identical across subgroups. The network structure invariance hypothesis infers similarity across subgroups in connectivity patterns, such as the extent of clustering. When significant, the invariance of specific edges in the network can be examined.

## Results

### Demographics

Participants were on average 17.44 years old (*SD* = 0.36), of which 949 were girls (56.6%) and 726 boys (43.4%). Discrimination was more prevalent among girls (*N* = 150; 73.2%) than boys (*N* = 55). Nearly a quarter of participants (23.9%) experienced moderate to severe depressive complaints (PHQ-9 score ≥ 10), and 20.9% experienced moderate to severe anxiety complaints (GAD-7 score ≥ 10), suggesting they may meet the criteria for a diagnosis on these mental illnesses. On average, participants experienced some level of stress or tension about once a week over the past month, and a moderate amount of stress from social relationships over the past year. Most participants had contact with at least one to three people in their neighborhood (79.4%) and perceived their neighborhood as safe (91.6%). This was similar in the sub-sample reporting discrimination, however, experiences of urban hassles (i.e. safety concerns, nuisance) were significantly higher among those who reported being discriminated against (*p* < 0.001). All mental health outcomes were, on average, significantly worse in participants who experienced discrimination compared to those who did not. Participants who felt discriminated against also reported lower social support and lower alcohol consumption compared to those who did not experience discrimination (*p* < 0.001). This lower alcohol consumption might be related to ethnic background, which was also reported most frequently as perceived motive for the discrimination. See Table 1a and Table 1b for sample details.

**Table 1:** **a**. Full sample descriptives of social safety perceptions including discrimination, objective neighborhood characteristics, mental health outcomes, and substance use (*N* = 1675). **b**. Sub-sample details of boys and girls who experienced discrimination (*N* = 205)

a			
	Boys (*N* = 726)	Girls (*N* = 949)	Total (*N* = 1675)
	*N* (%)	*N* (%)	*N* (%)
Ethnicity -			
Dutch	559 (77.0)	723 (76.2)	1282 (76.5)
Surinamese	17 (2.4)	42 (4.4)	59 (3.5)
Antillean	1 (0.1)	7 (0.7)	8 (0.5)
Turkish	5 (0.7)	9 (0.9)	14 (0.8)
Moroccan	15 (2.1)	25 (2.6)	40 (2.4)
Ghanaian	4 (0.6)	5 (0.5)	9 (0.5)
Western (other)	98 (13.5)	104 (11.0)	202 (12.1)
Non-Western (other)	27 (3.7)	34 (3.6)	61 (3.6)
Discrimination (yes)	55 (7.6)	150 (15.8) ↑	205 (12.2)
	**Mean (SD)**	**Mean (SD)**	**Mean (SD)**
UHI (0-45)	4.18 (3.14)	5.00 (3.53) ↑	4.65 (3.39)
MSPSS (21-84)	71.66 (9.48)	71.34 (10.72)	71.5 (10.2)
Objectively measured -			
Safety (z)	−0.016 (0.08)	−0.015 (0.08)	−0.015 (0.08)
Social cohesion (z)	−0.022 (0.06)	−0.022 (0.06)	−0.022(0.06)
Poverty (%)	6.95 (4.65)	7.05 (4.88)	7.01(4.78)
PHQ-9 (0-27)	4.84 (4.45)	7.93 (5.96) ↑	6.59 (5.57)
GAD-7 (0-21)	4.30 (3.55)	7.57 (4.98) ↑	6.15 (4.71)
Well-being (1-10)	7.66 (1.21)	7.20 (1.35) ↓	7.40 (1.31)
(Last 3 months)	**N (%)**	**N (%)**	**N (%)**
Alcohol use ≥ 1 time	517 (71.2)	659 (69.4)	1,176 (70.2)
Tobacco use ≥ 1 time	237 (32.6)	373 (39.4)	610 (36.4)
Cannabis use ≥ 1 time	270 (37.2)	276 (29.1) ↓	546 (32.6)

b			
	Boys (*N* = 55)	Girls (*N* = 150)	Total (*N* = 205)
	*N* (%)	*N* (%)	*N* (%)
Ethnicity -			
Dutch	28 (50.9)	84 (56.0)	112 (54.6)
Surinamese	6 (10.9)	16 (10.7)	22 (10.7)
Antillean	0	2 (1.3)	2 (1.0)
Turkish	1 (1.8)	4 (2.7)	5 (2.4)
Moroccan	4 (7.3)	8 (5.3)	12 (5.9)
Ghanaian	1 (1.8)	4 (2.7)	5 (2.4)
Western (other)	9 (16.4)	22 (14.7)	31 (15.1)
Non-Western (other)	6 (10.9)	10 (6.7)	16 (7.8)
Discrimination motive -			
Ethnic background	38 (69.1)	68 (45.3)	106 (51.7)
Physical appearance	29 (52.7)	60 (40.0)	89 (43.4)
Sexual preference	7 (12.7)	42 (28.0)	49 (23.9)
Clothing	10 (18.2)	29 (19.3)	39 (19.0)
Gender/sex	2 (3.6)	33 (22.0)	35 (17.1)
Religion	2 (3.6)	16 (10.7)	18 (8.8)
Other	6 (10.9)	15 (10.0)	21 (10.2)
	**Mean (SD)**	**Mean (SD)**	**Mean (SD)**
EDS (9-45)	20.36 (6.73)	21.22 (6.41)	20.99 (6.50)
UHI (0-45)	6.00 (4.21)	6.80 (4.03)	6.59 (4.09)
MSPSS (21-84)	69.61 (10.71)	67.25 (11.28)	67.89 (11.15)
PHQ-9 (0-27)	6.38 (5.37)	11.55 (7.30) ↑	10.17 (7.20)
GAD-7 (0-21)	5.49 (4.05)	10.38 (5.90) ↑	9.07 (5.88)
Well-being (1-10)	7.35 (1.49)	6.56 (1.64) ↓	6.77 (1.64)
(Last 3 months)	**N (%)**	**N (%)**	**N (%)**
Alcohol use ≥ 1 time	35 (63.6)	86 (57.3)	121 (59.0)
Tobacco use ≥ 1 time	18 (32.7)	54 (36.0)	72 (35.1)
Cannabis use ≥ 1 time	22 (40.0)	43 (28.7)	65 (31.7)

**a.** Descriptive statistics for main variables of interest including objective urban-neighborhood data. Variables marked with ↑ were significantly higher in girls than boys, and variables marked with ↓ were significantly lower in girls (*p* < 0.001 based on independent T-tests)

“ ≥ 1 time” refers to having used the substance at least once or more often in the prior 3 months. Range number of times alcohol use (0 = never; 1 = not in last 3 months; 2 = once; 3 = twice; 4 = 1–2 times per month; 5 = once per week; 6 = twice per week; 7 = 3 times per week; 8 = 4–5 times per week; 9 = daily); Range number of times tobacco use (0 = never; 1 = once in last 3 months; 2 = once per month; 3 = multiple times per month; 4 = once per week; 5 = multiple times per week; 6 = daily); Range number of times cannabis use (0 = never; 1 = not in last 3 months; 2 = once; 3 = 2–3 times; 4 = 4–10 times; 5 = 11–19 times; 6 = more than 19 times); **z** = positive/negative z-score means higher/lower than national average in the Netherlands

*UHI* urban hassles index, *MSPSS* multidimensional scale of perceived social support, *PHQ*-9 patient health questionnaire 9-items, *GAD*-7 general anxiety disorder 7-items

**b**. Variables marked with ↑ were significantly higher in girls than boys, and variables marked with ↓ were significantly lower in girls (*p* < 0.001 based on independent T-tests)

*EDS* every discrimination scale, indicating the experienced intensity of the discrimination in daily life; *Discrimination motive*: Note that participants were allowed to tick multiple boxes/motives for the discrimination they experienced. *UHI* urban hassles index, *MSPSS* multidimensional scale of perceived social support, *PHQ*-9 patient health questionnaire 9-items, *GAD*-7 general anxiety disorder 7-items

“ ≥ 1 time” refers to having used the substance at least once or more often in the prior 3 months. Range number of times alcohol use (0 = never; 1 = not in last 3 months; 2 = once; 3 = twice; 4 = 1–2 times per month; 5 = once per week; 6 = twice per week; 7 = 3 times per week; 8 = 4–5 times per week; 9 = daily); Range number of times tobacco use (0 = never; 1 = once in last 3 months; 2 = once per month; 3 = multiple times per month; 4 = once per week; 5 = multiple times per week; 6 = daily); Range number of times cannabis use (0 = never; 1 = not in last 3 months; 2 = once; 3 = 2-3 times; 4 = 4–10 times; 5 = 11–19 times; 6 = more than 19 times)

### Full Sample Network

**Fig. 1:**
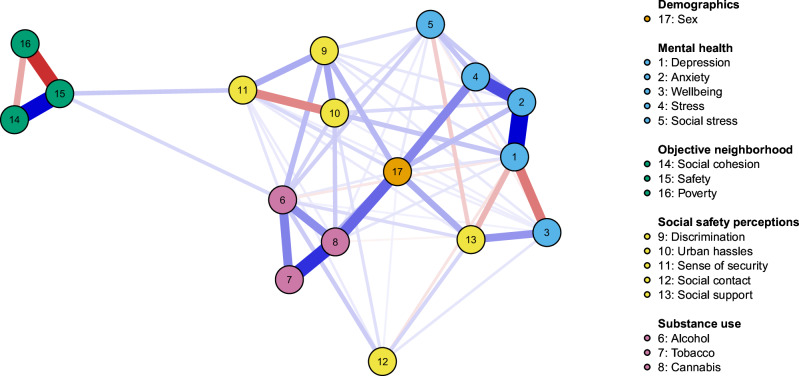
Full Sample Network. Each variable of interest represents a specific node within the network. Edges represent pairwise conditional associations, similar to partial correlation coefficients. Blue edges indicate positive associations, red edges indicate negative associations. The thickness and brightness of the edges reflect the strength of the association. In the legend, all subjectively measured social safety indicators are stated under “Social safety perceptions”. All objectively measured neighborhood social safety indicators are stated under “Objective neighborhood”

The Full-sample network is visualized in Fig. 1 and includes the dichotomous measure of discrimination. The network revealed clustering between the objective social safety measures and between the perceived social safety measures. Higher objective safety was directly connected with higher sense of security, a moderate positive link that suggests safer neighborhoods are consistently perceived as more secure by adolescents. This sense of security was strongly connected with fewer urban hassles and a lower likelihood of reporting discrimination. Substance use was directly linked to lower objective safety through alcohol use. Discrimination was negatively linked to alcohol use and sense of neighborhood security; but positively to experiencing more urban hassles, such as safety concerns and drug nuisance in one’s neighborhood. This moderate positive connection highlights that discrimination co-occurs with everyday stressors in the urban environment. Perceiving more urban hassles was also directly associated with higher likelihood of increased cannabis use and depressive symptoms; showing that those who experience more urban hassles more often use cannabis and more often report depressive symptoms. There was a moderate direct connection between discrimination and higher levels of symptoms of depression and anxiety; and lower levels of well-being. This suggests that discrimination might be more consistently related to mental health than to substance use behaviors.

**Fig. 2:**
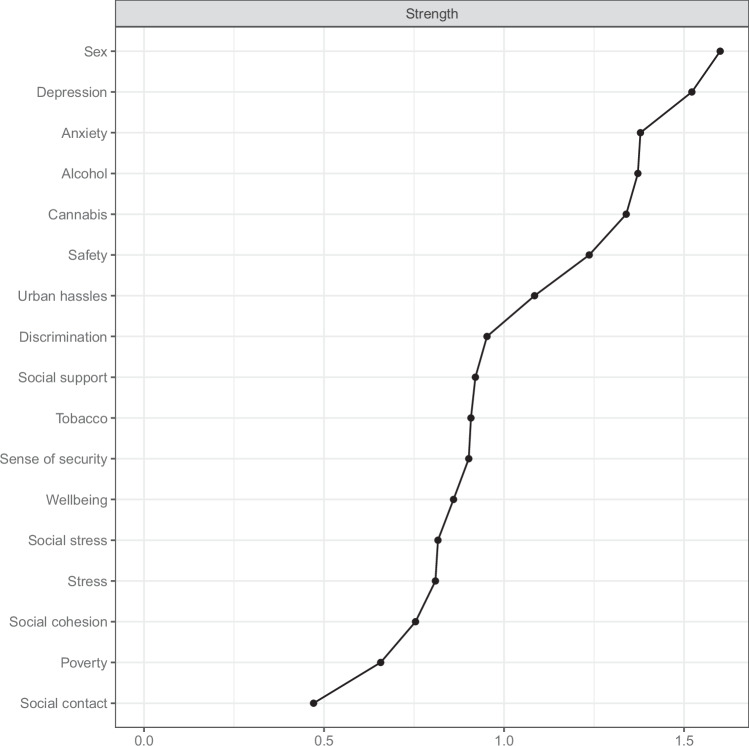
Strength Centrality (Full Sample). Strength centrality identifies the most influential or connected variables in the network. It is computed as the weighted number and strength of all edges connected to a given node, highlighting which variables play a key role in the system as a whole

**Fig. 3:**
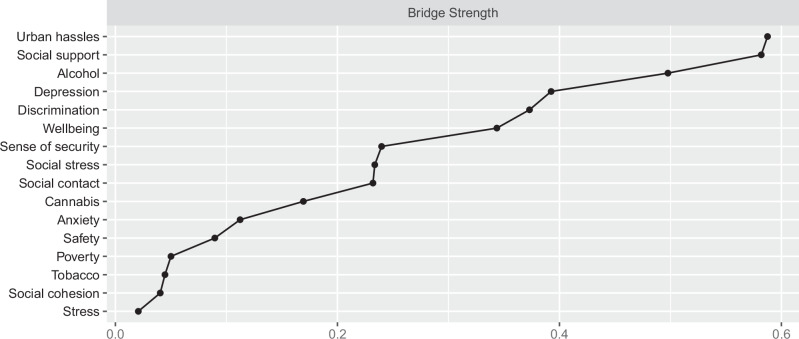
Bridge Centrality (Full Sample). Bridge centrality identifies variables that serve as connections between different groups or clusters of variables, indicating potential pathways through which associations spread across domains

The three strongest nodes in the network were sex, symptoms of depression, and symptoms of anxiety (Fig. 2). The three strongest connecting nodes—urban hassles, social support, and alcohol use—serve as bridges between objective neighborhood characteristics, social safety perceptions, mental health, and substance use (Fig. 3). These bridge nodes highlight how everyday stressors and social resources connect neighborhood context with psychological and behavioral outcomes. Bootstrapping analysis confirmed good model stability (see Supplementary Materials Fig. S1). Case-dropping analysis showed a Case Stability-coefficient of 0.82, indicating robust stability of strength centrality even when only 10% of the sample remained (Fig. S2).

### Boys vs. Girls

The full-sample network highlighted the central role of sex, hence, a network comparison test was performed comparing boys and girls. This test revealed significant differences in global strength (*p* = 0.008) but not in network structure (*p* = 0.078), making further testing of specific edges unnecessary (van Borkulo et al., 2023). The higher global strength in the girls’ network suggests overall stronger connections among social safety perceptions, mental health, and substance use compared to the boys’ network (Fig. S5).

### Sub-Sample Network

**Fig. 4:**
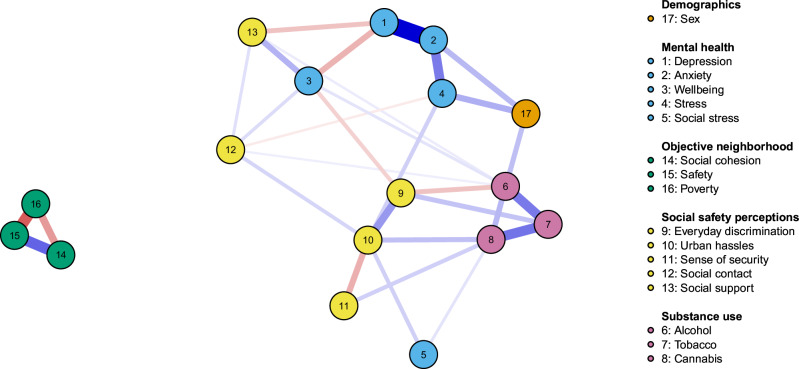
Sub-Sample Discrimination Network. The sub-sample consists of participants who reported that they felt discriminated against. Each variable of interest represents a specific node within the network. Edges represent pairwise conditional associations, similar to partial correlation coefficients. Blue edges indicate positive associations, red edges indicate negative associations. The thickness and brightness of the edges reflect the strength of the association. In the legend, all subjectively measured social safety indicators are stated under “Social safety perceptions”. All objectively measured neighborhood social safety indicators are stated under “Objective neighborhood”

The network including only participants who felt discriminated against is visualized in Fig. 4. Objective safety, poverty, and social cohesion had no direct link with social safety perceptions, mental health, and substance use. The score indicating recurrence of everyday discrimination replaced the dichotomous variable, and had a central position in the network. Everyday discrimination was closely linked to neighborhood perceptions, specifically more urban hassles experiences and lower sense of security within one’s neighborhood. This strong positive link suggests that everyday discrimination and urban hassles are closely interconnected and may jointly play a central role in adolescents’ lived experiences. Everyday discrimination was also indirectly connected to higher levels of depression symptoms, anxiety symptoms, and stress through pathways involving lower well-being and social support. A strong positive link was found between everyday discrimination and smoking, which was closely related to cannabis use. This showed that adolescents who experienced greater everyday discrimination were more likely to engage in smoking. Conversely, everyday discrimination showed a negative relationship with alcohol use. Overall, perceived urban hassles seem strongly interlinked with everyday discrimination, sense of security, stress, and social stress. A noteworthy finding was the direct positive association between sense of security and cannabis use, as well as the positive link between urban hassles and social contact. Though not anticipated based on prior literature, this finding may point to complex dynamics between perceived safety, experimentation with substances, and neighborhood interactions.

**Fig. 5:**
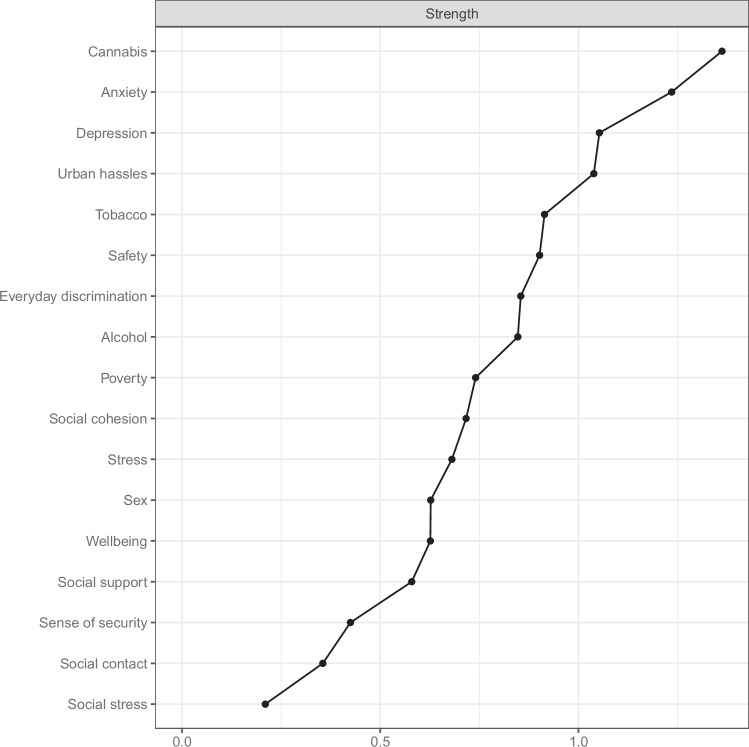
Strength Centrality (Sub-Sample Discrimination). Strength centrality identifies the most influential or connected variables in the discrimination sub-sample network. It is computed as the weighted number and strength of all edges connected to a given node

**Fig. 6:**
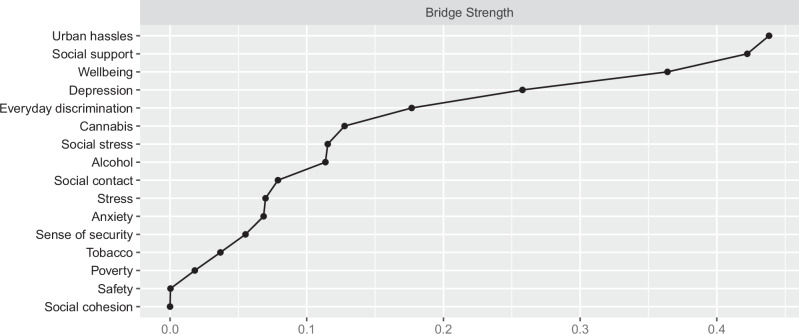
Bridge Centrality (Sub-Sample Discrimination). Bridge centrality identifies variables that serve as connections between different groups or clusters of variables in the discrimination sub-sample network

The top three nodes with highest strength centrality were cannabis, anxiety, and depression (Fig. 5). The three strongest bridge nodes were urban hassles, social support, and well-being (Fig. 6). These bridge nodes emphasize how stressors and protective factors jointly connect everyday discrimination experiences with mental health and substance use. Bootstrapping and case-dropping results reveal moderate model stability estimates with a Case Stability-coefficient of 0.50, indicating moderate stability of centrality estimates in this subgroup (Fig. S3, S4).

## Discussion

Research on adolescent mental health has increasingly highlighted the importance of contextual stressors, such as discrimination and neighborhood social safety, yet these domains are often examined separately. Few studies have integrated objective and subjective neighborhood indicators with experiences of discrimination to understand their combined impact on mental health and substance use. Addressing this gap requires approaches that capture the complex interrelations among these factors. This study contributes to this effort by applying a network analytical approach in a Dutch adolescent sample, providing a general overview of how discrimination and social safety are linked to mental health and substance use. Building on this rationale, the following section highlights the key findings from the network analysis.

### Key Findings From the Network Analysis

This study revealed key connections between mental health, discrimination, social safety, and substance use in late adolescents. The full-sample network highlighted the central role of discrimination, urban hassles, and sense of neighborhood security in linking objective neighborhood social safety to mental health outcomes. Discrimination was negatively associated with the sense of neighborhood security and alcohol use, while positively connected to perceived urban hassles and symptoms of anxiety and depression. Cannabis and tobacco use were positively associated with both urban hassles and having experienced discrimination, whereas alcohol use was negatively associated with everyday discrimination. Greater everyday discrimination held a central role, and was interrelated with more mental health problems through lower well-being, and lower levels of social support. There were direct connections between greater everyday discrimination and more urban hassles experiences and cannabis use.

### Substance Use Patterns in Relation to Discrimination

Network analysis highlighted notable patterns in the relationships between mental health, discrimination, social safety, and substance use. While previous research has established positive associations between discrimination and mental health problems (Bardol et al., 2020; Benner et al., 2018), the findings add nuance by showing positive links between discrimination and cannabis use and smoking, yet a negative link with alcohol use. This divergence in substance use behaviors might suggest varied substance use motives among adolescents facing discrimination, which requires further exploration. Substance use among adolescents is not solely driven by coping motives; other factors, such as social or enhancement motives are more prominent in young individuals (Kuntsche et al., 2006), and these motivations may vary depending on the specific substance (Cooper et al., 2016). For instance, while some individuals may use substances to alleviate stress, others may engage in substance use for social bonding or sensation-seeking. The lower risk of alcohol use may also reflect cultural and religious values that discourage alcohol consumption, particularly among adolescents from ethnic minority backgrounds who experience ethnic-based discrimination (Evans-Polce et al., 2020; Visser et al., 2017). As such, cannabis use and smoking behaviors may, in some contexts, be perceived as less culturally restricted than alcohol use. However, these associations are likely multifactorial and may reflect a combination of coping motives, peer dynamics, cultural or religious norms, and broader contextual influences. For instance, meta-analytic evidence demonstrates consistent detrimental effects of racial/ethnic discrimination across adolescent well-being domains, including depressive symptoms, externalizing behaviors, and substance use (Benner et al., 2018). In the Dutch school context, teacher discrimination has been shown to predict externalizing behavior among Muslim youth (van Bergen et al., 2021). Findings from the HELIUS study in Amsterdam further indicate that ethnic discrimination was differentially associated with smoking and alcohol use across minority groups with specific ethnic backgrounds (Visser et al., 2017). Together, these examples illustrate that coping responses to discrimination vary across ethnic groups, substance types, contexts, and behaviors. The current findings point to complex, substance-specific dynamics that warrant further investigation.

### Ethnic Background and Perceptions of Discrimination

Within the current sample, discrimination based on ethnic background and physical appearance was reported most frequently. Internationally, ethnic-based discrimination has been identified as a significant predictor of increased psychological distress and reduced well-being across all age-groups (Bardol et al., 2020), with adolescents facing the highest risks due to their developmental vulnerability (Mello, 2024). It should be noted that some youth who reported ethnic-based discrimination were categorized as having a Dutch or Western ethnic background. Ethnicity in the ABCD cohort was determined based on the maternal grandmother’s country of birth. Some adolescents may have a mixed ethnic background, for example when the mother was Dutch and the father had a different migration background. These experiences may reflect subjective interpretations of exclusion based on cultural, linguistic, or appearance-related cues, and highlight the need for a refined understanding of how adolescents perceive and label discrimination.

### Sex Differences

Girls, specifically, may face increased vulnerability due to the combination of societal expectations (i.e. physical appearance; intellectual pressure; and financial independence) and their tendency to be more emotionally responsive to contextual stressors (Haraldsson et al., 2011). As expected, girls reported higher depression and anxiety symptoms, reflecting well-documented sex differences in emotional vulnerability during adolescence (Boer et al., 2022; Yoon et al., 2023). Notably, sex was a central node, with discrimination being more prevalent among girls. The girls’ network showed denser connections between social safety perceptions, mental health, and substance use, possibly suggesting a heightened sensitivity to social and environmental stressors (Haraldsson et al., 2011). This interaction between external pressures and internal sensitivity can create a more challenging environment to navigate in daily life (Andriessen et al., 2020; Boer et al., 2022). Nonetheless, the potential role of residential discrimination in adolescent mental health remains understudied, particularly in relation to structural feelings of isolation and distress among minority youth (Acker et al., 2023). Understanding social safety in residential environments within broader sociocultural contexts is therefore essential.

### Subjective vs. Objective Neighborhood

Importantly, analyses indicated that discrimination was closely linked to perceived neighborhood social safety, including urban stressors and security concerns. Although the cross-sectional design does not allow for causal inferences, these results may be interpreted as discrimination disrupting adolescents’ sense of security and stability in their living environments, potentially amplifying its impact on mental health and behavior (Midouhas et al., 2021). However, the opposite may also be true: adolescents who perceive their environment as unsafe may also be more likely to perceive discrimination. Such perceptions could, at least in part, reflect an increased sensitivity to social and environmental stressors, shaped by their mental health status (Midouhas et al., 2021; Visser et al., 2021). Interestingly, objective neighborhood characteristics, such as poverty levels and social cohesion, appeared to play a less direct role in adolescents’ perceptions of social safety—particularly regarding discrimination and overall mental well-being. This suggests that subjective experiences and interpretations may be more prominent in these associations than physical neighborhood factors alone.

### Discrimination and Mental Health

Discrimination in daily life has been recognized in existing literature as a significant chronic social stressor, with the potential to exacerbate mental health challenges among adolescents (Benner et al., 2018; Zhao et al., 2024). Persistently feeling unsafe or unwelcome in one’s own neighborhood alongside discriminatory experiences may contribute to heightened stress and reduced well-being, creating a pathway toward more severe psychological distress, including depression and anxiety symptoms (Baranyi et al., 2021; Lavner et al., 2022). This aligns with the current findings, demonstrating strong associations between discrimination, neighborhood safety, urban hassles, and reduced social support. Importantly, social support emerged as a critical protective factor, bridging the relationship between mental health, social safety, and substance use. Prior research suggests that the adverse effects of environmental stressors often manifest initially as reduced well-being and increased stress experience, with social support serving as a buffer that mitigates these early impacts (Koelen et al., 2022; Rueger et al., 2016). However, in the absence of adequate support, these stressors may escalate over time into chronic mental health conditions (Szkody & McKinney, 2019; Wang et al., 2018). Discrimination is not only a personal experience but also a broader social determinant that can elevate stress levels across multiple life domains, including friendships, family, school/work, and major life events (Zhao et al., 2024). Such experiences may disrupt adolescents’ sense of security within their residential communities, contributing to increased internal stress feelings and diminished well-being. Drawing on Stress Response Theory (Godoy et al., 2018), adolescents who repeatedly experience discrimination may develop heightened stress reactivity. Chronic exposure to social stressors can keep the body and mind in a prolonged state of hypervigilance (Godoy et al., 2018), leading to emotional dysregulation and increased vulnerability to mental health disorders (Lavner et al., 2022; Priest et al., 2013). Facing persistent unfair treatment may undermine adolescents’ sense of environmental safety, potentially increasing their reliance on harmful behaviors like substance use (Dai et al., 2024; Gerrard et al., 2012; Lei et al., 2021). Strengthening social support systems may help mitigate these effects by fostering a collective sense of security and resilience among adolescents.

### Future Research Directions

The analysis indicates that discrimination may undermine mental health by weakening social support and reducing well-being. The observed connections between perceptions of discrimination, urban hassles, and reduced neighborhood safety raise important questions about the role of community dynamics. Although this study explored associations rather than establishing causal relationships, these findings highlight areas requiring further exploration. As said, it is important to consider that adolescents experiencing higher levels of anxiety and depression symptoms may be more likely to perceive their environment as unsafe and discriminatory. This possibility suggests a bidirectional relationship, where poor mental health may both result from and contribute to heightened sensitivity to one’s living environment. Further research is needed to disentangle these complex interactions and explore how these dynamics uniquely affect adolescents of different demographic backgrounds. Notably, although adolescent girls appeared to be more sensitive to discrimination as a social stressor, few studies have examined sex differences in the intersection of discrimination and neighborhood social safety perceptions. Much of the existing research on discrimination has focused on schools, workplaces, and peer interactions (Montoro et al., 2021; van Bergen et al., 2021); while the role of discrimination in residential social safety remains an underexplored area. Longitudinal studies are particularly needed to assess the long-term direction of effects of discrimination on adolescents’ perceptions of social safety and how sense of neighborhood insecurity and mental health may co-develop over time. Specifically, to provide insights that can inform hypothesis-driven longitudinal models examining how social safety in the living context predict the course of mental health and substance use problems during late adolescence. Additionally, future research should investigate protective factors, such as inclusive community structures and strong social support systems, that may help mitigate the detrimental effects of discrimination. Strengthening these networks and fostering a sense of belonging within residential environments could represent a promising avenue for intervention, ultimately enhancing adolescent well-being.

### Preliminary Implications

The findings suggest that experiences of discrimination are closely linked to perceptions of neighborhood safety and everyday urban stressors. From a practical perspective, this highlights the importance of fostering inclusive and supportive community environments. Municipalities and local organizations could consider initiatives that actively reduce discrimination, strengthen social support networks, and address safety concerns in public spaces. Possible directions include expanding accessible public spaces that facilitate positive community interaction, strengthening measures that enhance feelings of safety, and supporting initiatives that build trust and engagement among residents. Educational institutions, community centers, and local organizations can contribute by organizing activities that foster social cohesion and bring adolescents and neighbors together. At the municipal level, policies may also proactively address environmental stressors and safety concerns, for example through stricter regulation of drug-related nuisance, improved upkeep of shared spaces, and targeted interventions in neighborhoods facing high levels of social disorganization. Such efforts may help improve adolescents’ sense of security and well-being, while also contributing to healthier and more cohesive urban communities. Nonetheless, these implications should be interpreted with caution, given the correlational nature of the study, but they may help guide future intervention research and community strategies.

### Strengths and Limitations

This study offers valuable insights into the complex relationships between mental health, social safety, discrimination, and substance use among adolescents. The use of network analysis allowed for an exploration of the connections between these factors and a detailed visualization of these connections, highlighting key factors such as discrimination, urban hassles and social support. This network approach made it possible to uncover how depressive and anxiety symptoms, substance use, experienced social safety and discrimination are interrelated within adolescents’ residential environments. By identifying central and bridging nodes—such as urban hassles and social support—we were able to visualize how stressors and protective factors co-occur and potentially reinforce each other, offering insights that may remain hidden in traditional regression-based models. By combining objective and perceived measures, this study gives a balanced view of how social safety in adolescents’ living environments is linked to feelings of discrimination. Moreover, the inclusion of objective indicators offers the opportunity to assess whether subjective perceptions are supported by contextual realities, thereby strengthening the interpretability of the findings. These findings are an important first step in exploring discrimination as a key part of creating safe neighborhoods that support adolescent mental health and well-being. However, the cross-sectional design limits the ability to establish causal relationships or determine the direction of connections between mental health and social safety perceptions in the context of discriminatory experiences. Moreover, other unmeasured factors such as peer influences, family dynamics, or broader cultural norms may also contribute to these relationships. Future longitudinal research could provide deeper insights into these dynamic processes over time. Additionally, self-reported data may be subject to bias, including underreporting or misinterpretation of sensitive experiences such as discrimination. Most study participants were of Dutch origin and from relatively high socioeconomic positioned families, which may have led to an underestimation of discrimination and could limit the generalizability of the findings to more diverse populations. Moreover, ethnicity in the ABCD cohort was categorized based on the maternal grandmother’s country of birth, using predefined groups (e.g., Dutch, Surinamese, Turkish, Moroccan, Other Western, Other Non-Western). Most participants had a Dutch or Western ethnic background (69.7%), while 30.3% had a non-Western background, but multiethnic identities could not be captured with the available data. This limitation may therefore oversimplify the ethnic composition of the sample, potentially obscuring intersectional experiences of discrimination and the generalizability of findings to more diverse or intersectional populations. Data on the specific context (i.e. neighborhood, peer network, or school/work) of the experienced discrimination was not available. The geographical context of the Dutch population sample may also limit the generalizability to areas with different levels of urbanization and cultural diversity. While the ABCD cohort is a population-based cohort, participation relies on voluntary response. As such, the findings may not be fully generalizable to all Dutch adolescents. Nonetheless, the sample remains socio-demographically diverse and includes substantial representation of ethnic minority youth, which strengthens the relevance of the findings for urban adolescent populations. Finally, while this research explored a wide range of social environmental stressors, there may have been other unforeseen influences that can impact the overall mental state of adolescents, for instance timely help-seeking possibilities, socio-cultural influences, and policy changes across the COVID-period.

## Conclusion

Discrimination in residential contexts has been an underexplored factor in adolescent mental health, particularly in relation to perceptions of neighborhood social safety. By integrating objective and perceived measures, the study addressed this gap and revealed that discrimination is closely linked to urban hassles, reduced well-being and social support, and heightened symptoms of anxiety and depression. These relationships appeared more pronounced among girls, possibly reflecting greater sensitivity to social stressors such as discrimination and safety concerns. These findings underscore the importance of considering discrimination as a central social stressor in late adolescence and highlight its role in both mental health and substance use domains. They also lay a foundation for future longitudinal research to clarify whether discrimination alters social safety perceptions and mental health trajectories over time, or whether pre‑existing vulnerabilities shape adolescents’ interpretations of their environments. Addressing discrimination and fostering inclusive, supportive communities may be essential for promoting adolescent mental health and creating socially safe neighborhoods.

## Supplementary information

Below is the link to the electronic supplementary material.

Supplementary Information

## Data Availability

The data that support the findings of this study are not publicly available due to privacy and ethical restrictions, specifically participant confidentiality and data protection regulations.
