# Supplementary material for: The Role of Discrimination in Social Safety and its Interplay with Adolescents’ Mental Health and Substance Use: A Network Perspective
Source: J Youth Adolesc. 2026 Mar 30;55(7):1678–96. doi: 10.1007/s10964-026-02344-7 (PMC13328147; doi:10.1007/s10964-026-02344-7)
Supplement: Supplementary file 1 — Supplementary Information [file 10964_2026_2344_MOESM1_ESM.pdf]

Fig. S1. Non-Parametric Bootstrapping (Full Sample)

Note: Bootstrapping analysis confirmed good model stability,
as confidence intervals around edge weights showed minimal deviation from the sample estimates.
The analysis checks for deviations between the bootstrapped mean and the sample mean for each edge.

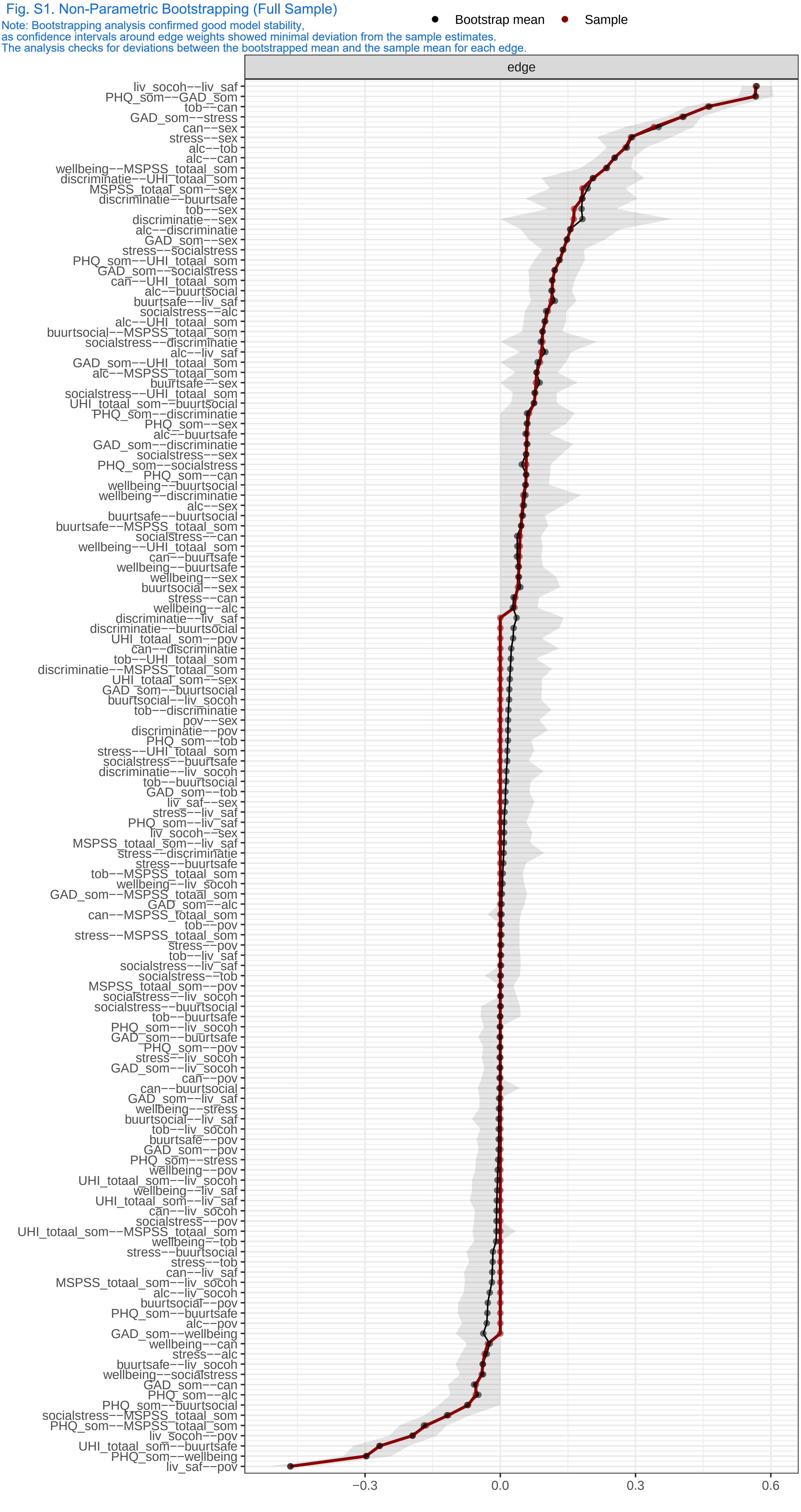

Fig. S2. Case Stability (Full Sample)

Note: Case-dropping analysis indicated preserved stability of strength centrality,
with the correlation stability coefficient remaining robust even when only 10% of the sample was retained.
A coefficient at or above 0.5 is deemed acceptable.

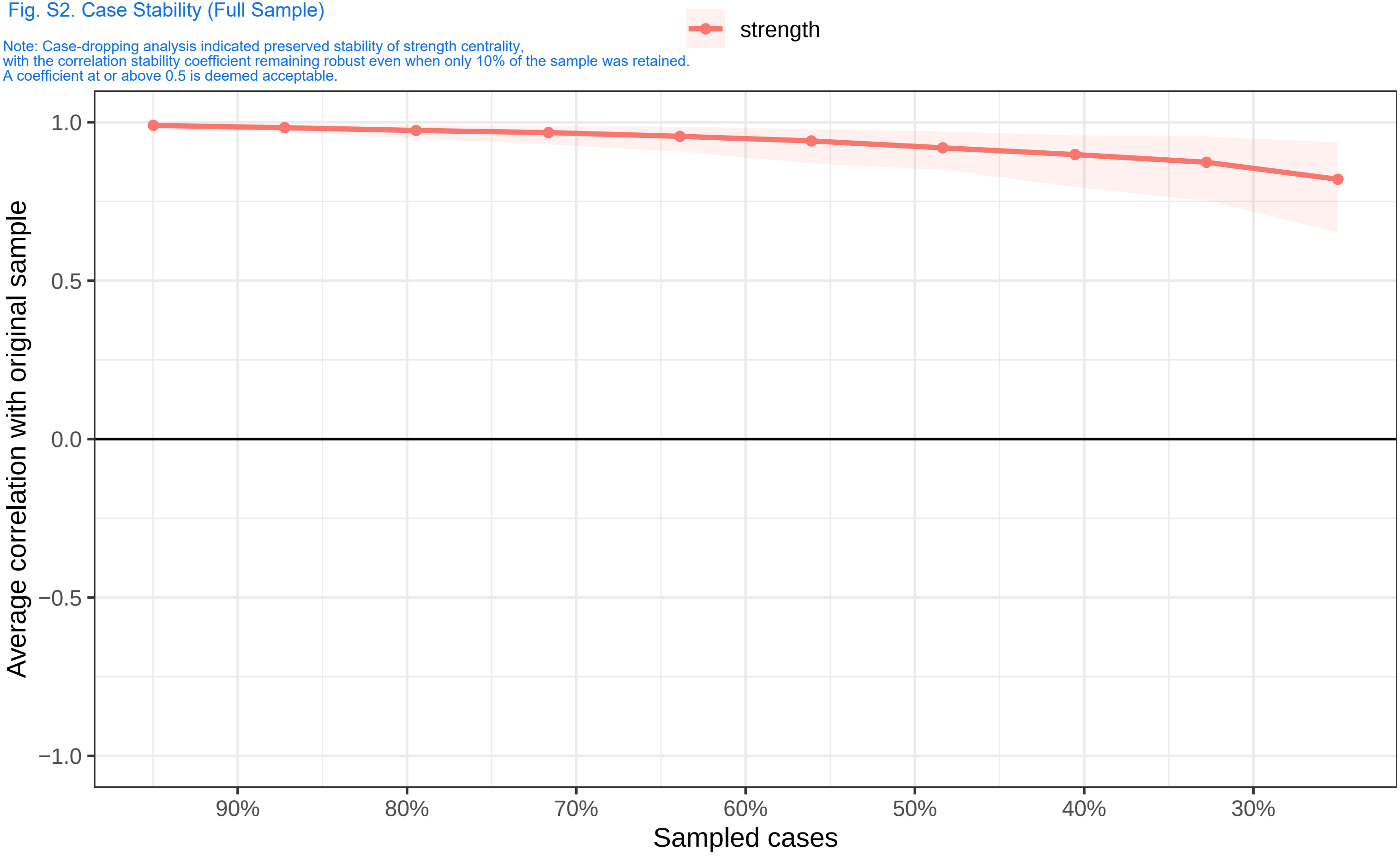

Fig. S3. Non-Parametric Bootstrapping (Sub-Sample Discrimination)

Note: Bootstrapping analysis confirmed good model stability,
as confidence intervals around edge weights showed minimal deviation from the sample estimates.
The analysis checks for deviations between the bootstrapped mean and the sample mean for each edge.

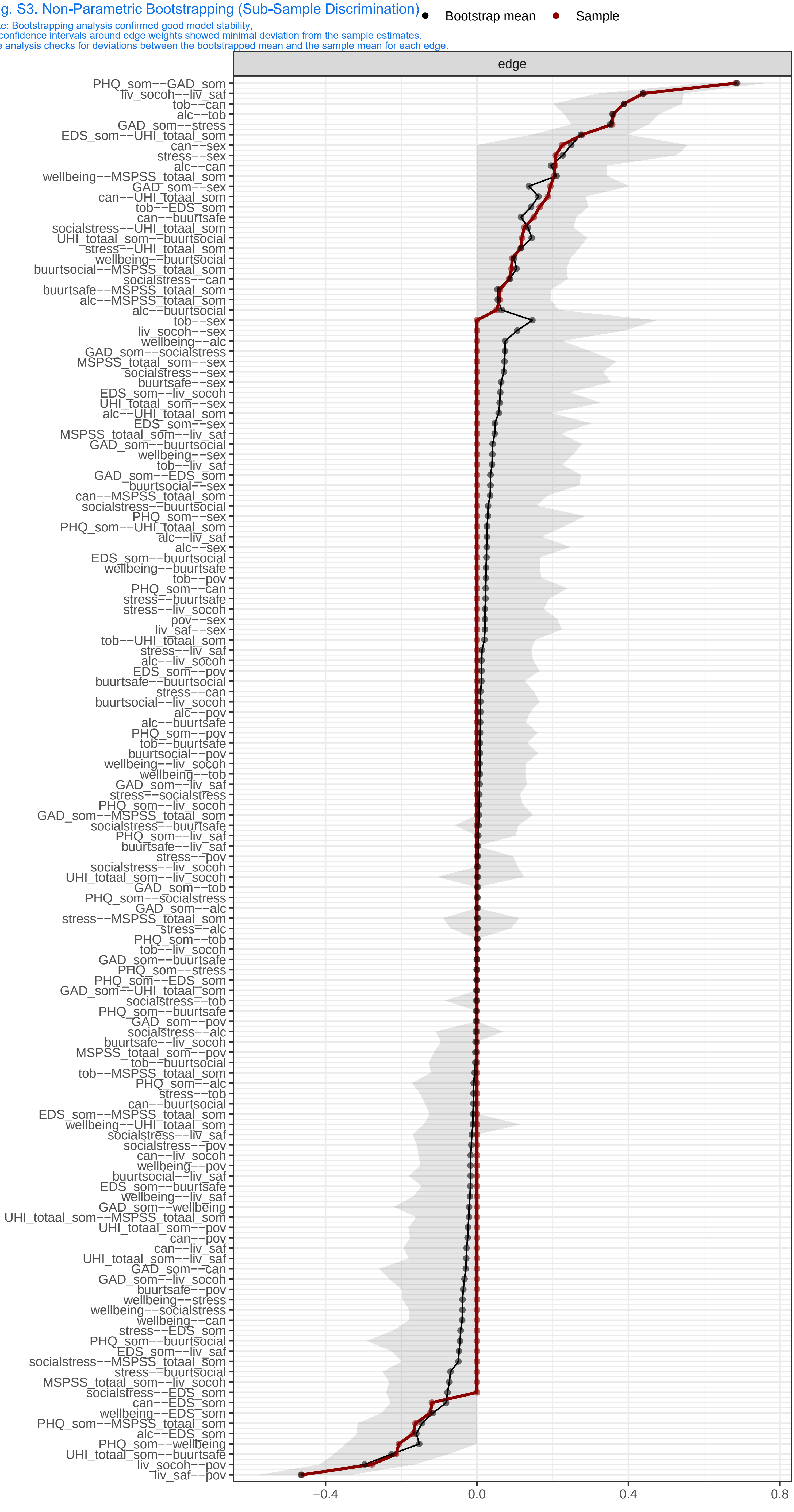

Fig. S4. Case Stability (Sub-Sample Discrimination)

Note: Case-dropping analysis indicated preserved stability of strength centrality,
with the correlation stability coefficient remaining robust even when only 10% of the sample was retained.
A coefficient at or above 0.5 is deemed acceptable.

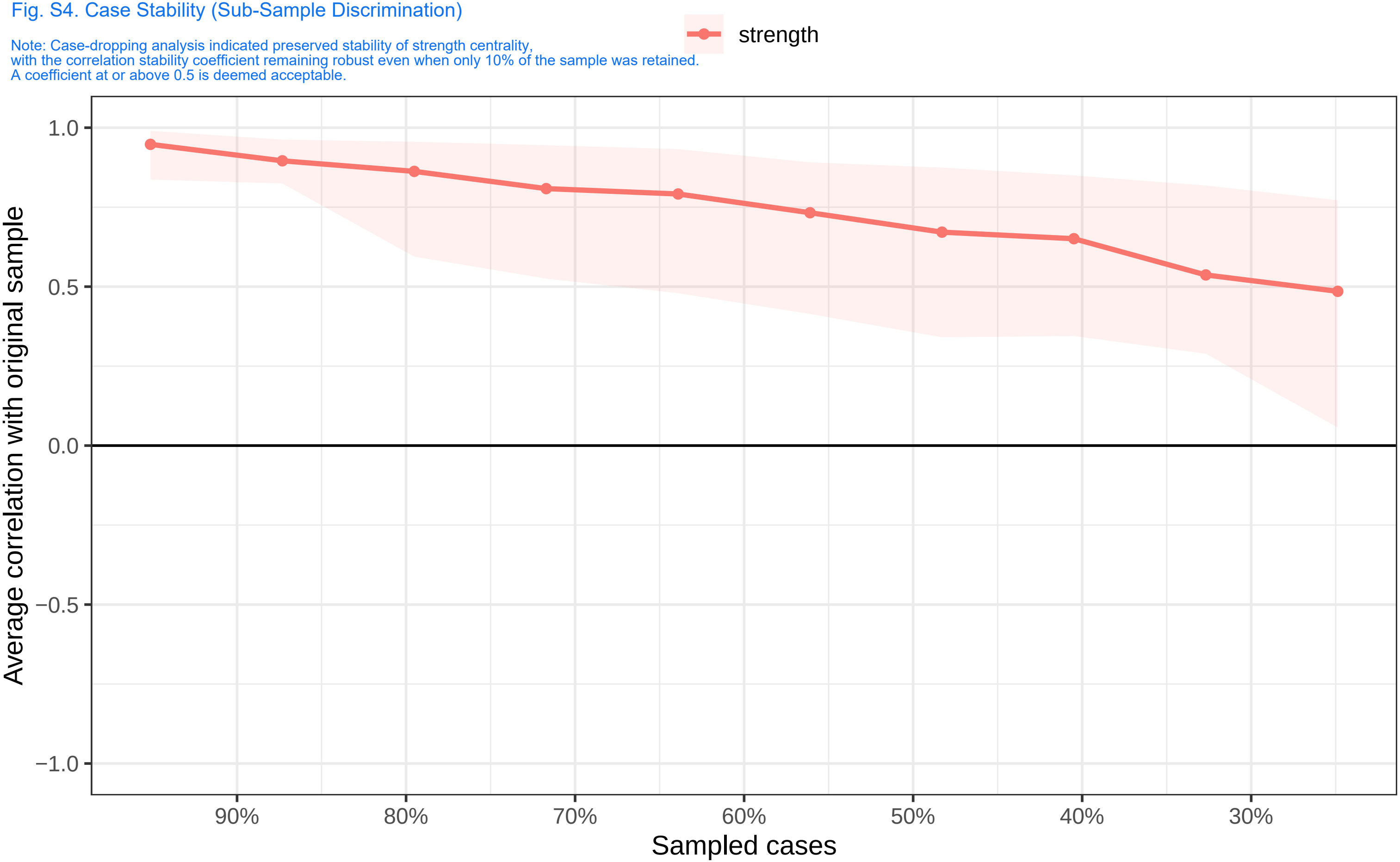

Fig. S5. Network Comparison: Boys vs. Girls

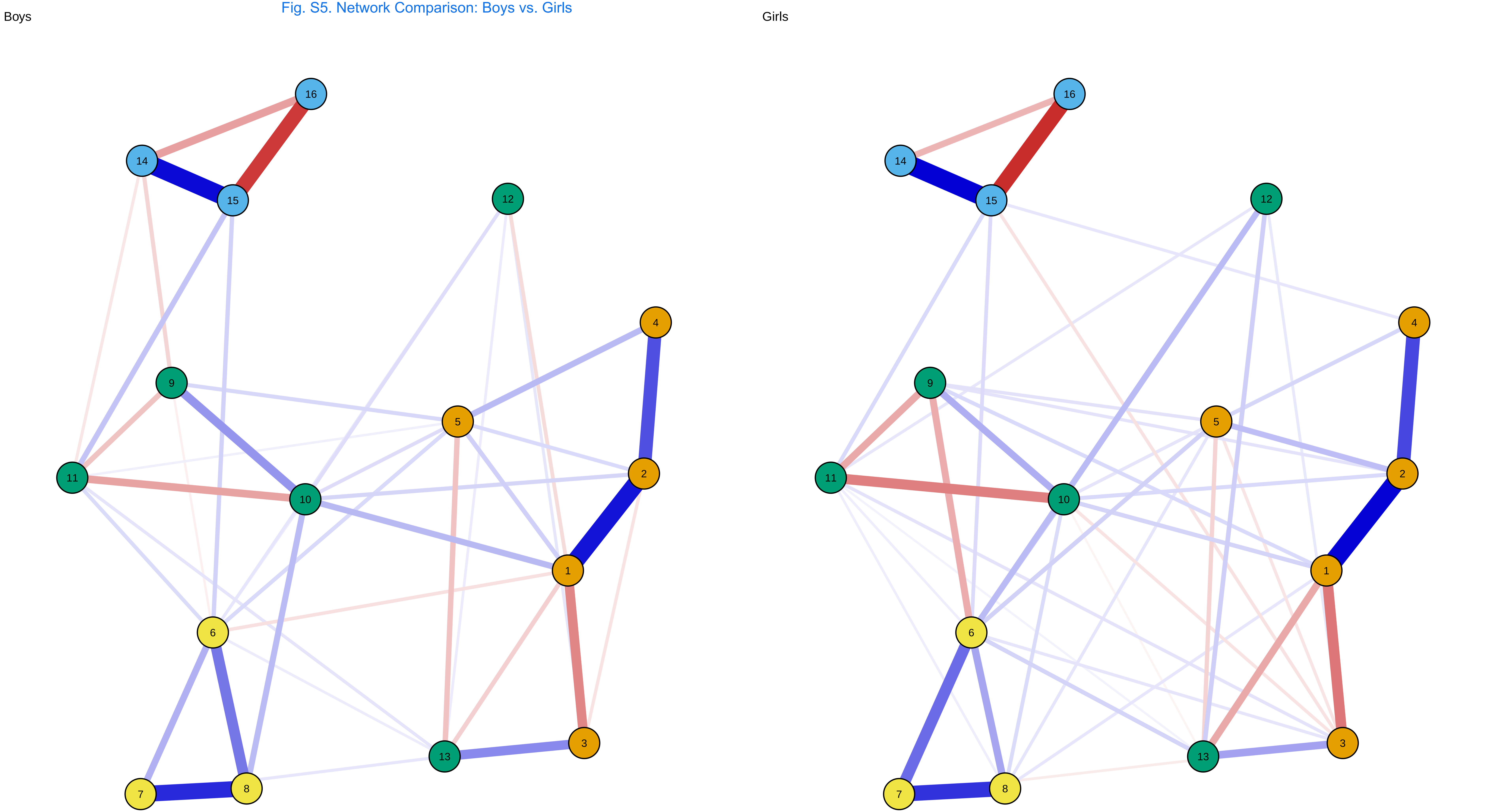

Note:
Each variable of interest represents a specific node within the network.
Edges represent pairwise conditional associations, similar to partial correlation coefficients.
Blue edges indicate positive associations, red edges indicate negative associations.
The thickness and brightness of the edges reflect the strength of the association.
